# Neural bases for attenuation of morphine withdrawal by Heantos-4: role of *l*-tetrahydropalmatine

**DOI:** 10.1038/s41598-020-78083-x

**Published:** 2020-12-04

**Authors:** Soyon Ahn, Maya O. Nesbit, Haiyan Zou, Giada Vacca, Peter Axerio-Cilies, Tran Van Sung, Anthony G. Phillips

**Affiliations:** 1 Department of Psychiatry, University of British Columbia, Vancouver, V6T 2A1 Canada; 2 Institute of Chemistry, Vietnam Academy of Science and Technology, Hanoi, Vietnam

**Keywords:** Addiction, Translational research, Animal disease models

## Abstract

Severe withdrawal symptoms triggered by cessation of long-term opioid use deter many individuals from seeking treatment. Opioid substitution and α2-adrenergic agonists are the current standard of pharmacotherapy for opioid use disorder in western medicine; however, each is associated with significant complications. Heantos-4 is a non-opioid botanical formulation used to facilitate opioid detoxification in Vietnam. While ongoing clinical use continues to validate its safety and effectiveness, a mechanism of action accounting for these promising effects remains to be specified. Here, we assess the effects of Heantos-4 in a rat model of morphine-dependence and present evidence that alleviation of naloxone-precipitated somatic withdrawal signs is related to an upregulation of mesolimbic dopamine activity and a consequent reversal of a hypodopaminergic state in the nucleus accumbens, a brain region implicated in opioid withdrawal. A central dopaminergic mechanism is further supported by the identification of *l*-tetrahydropalmatine as a key active ingredient in Heantos-4, which crosses the blood–brain barrier and shows a therapeutic efficacy comparable to its parent formulation in attenuating withdrawal signs. The anti-hypodopaminergic effects of *l*-tetrahydropalmatine may be related to antagonism of the dopamine autoreceptor, thus constituting a plausible mechanism contributing to the effectiveness of Heantos-4 in facilitating opioid detoxification.

## Introduction

Increased access to potent pain-relieving opioids, through legal or illicit means, is linked to an unprecedented rise in opioid use disorder (OUD) and opioid-related deaths in developed and developing nations^1^. Opioid detoxification is a critical first step in transitioning to long-term management of OUD. The cessation of use, however, triggers debilitating affective and somatic withdrawal signs that can last for several days to weeks and difficulty enduring this period may contribute to relapse^2,3^. Even the anticipation of these negative states serves as a major deterrent against seeking clinical treatment. Currently, the main mode of medically-assisted detoxification involves substitution therapy with long-acting opioids (e.g., methadone, buprenorphine) alone or in combination with µ-opioid antagonists (e.g., naltrexone)^2,4^. Unfortunately, these opioids also carry the risk of misuse and diversion to illicit use. The recent FDA approval of the α2-adrenergic agonist lofexidine to facilitate opioid withdrawal is welcome news^5^; however, its utility is limited by hypotensive side-effects that contribute to further lowering of compliance rates^6^.

Medicinal plants traditionally used in Asia to treat pain, anxiety and gastrointestinal discomfort are gaining recognition as non-opioid treatment options for heroin dependence^7–9^; notably, some demonstrate an efficacy comparable to lofexidine but with fewer side-effects. Heantos-4 is a patented botanical formulation invented in Vietnam over 30 years ago to facilitate detoxification from opioids^10^. Formal toxicological and clinical trials conducted in Vietnam, with support from the United Nations Development Programme and other international institutions, as well as ongoing clinical use continue to validate the effectiveness and safety of Heantos-4 as a detoxification treatment^10,11^. However, a mechanism of action that accounts for these promising effects remains to be specified.

A key pathological feature of chronic exposure to opioids is a persistent dysfunction of the mesolimbic dopamine (DA) system^12–15^, with discontinuation resulting in a hypodopaminergic state^16^. In individuals that have undergone heroin detoxification, imaging studies reveal a decrease in striatal D2/3 receptor binding and a reduction in presynaptic DA release^17^. These observations are corroborated by preclinical reports of withdrawal-associated reductions in firing rates of DA neurons in the midbrain along with attenuated DA efflux in the basal forebrain^18–24^. Based on these data, normalization of hypodopaminergia has been proposed as a pharmacotherapeutic strategy to facilitate cessation of opioid use^2,16,25^. Indeed, this therapeutic rationale is consistent with the findings of our initial preclinical assessment of Heantos-4, which confirmed in morphine-dependent rats the alleviation of naloxone-induced hypodopaminergia and significant attenuation of somatic withdrawal measures^19^. Similar outcomes were also observed with the α2-adrenergic agonist clonidine^18^, suggesting that normalization of DA function may be achieved even by indirect effects on the DA system.

The present investigation was guided by the hypothesis that Heantos-4 facilitates detoxification from opioids through direct modulation of the DA system. Employing in vivo microdialysis, we first conducted a series of neuropharmacological investigations in the nucleus accumbens (NAc), a key region of the mesocorticolimbic DA system implicated in opioid reward^13^ as well as the expression of somatic and aversive features of opioid withdrawal^26,27^. The first experiment, using morphine-dependent rats, examined if Heantos-4 could reverse a hypodopaminergic state in the NAc precipitated by the µ-opioid antagonist naloxone after its onset and concurrently alleviate somatic correlates of withdrawal. We next considered the possibility of a therapeutic mechanism of action directed at the DA autoreceptor, which has a critical role in moderating DA transmission especially following exposure to opioids and other drugs of abuse^28^. Presynaptic DA autoreceptors (including the D2S short isoform and D3 subtypes) orchestrate the synthesis, storage and release of DA^29–32^. At appropriate low doses, D2/D3 agonists augment auto-inhibition of DA release, thereby provoking a hypodopaminergic state; in contrast, D2 antagonists block feedback inhibition, in effect, stimulating DA release^33–36^. It is conceivable that the anti-hypodopaminergic effects of Heantos-4 similarly involves blockade of the DA autoreceptor and a consequent upregulation of DA release (i.e., a DA autoreceptor antagonist). Thus, a second experiment examined a putative DA autoreceptor antagonist property of Heantos-4 against the hypodopaminergic effects of the D2/3 agonist quinpirole.

Heantos-4 is composed of extracts from twelve medicinal plants (see Table 1 in ref.^19^), the qualitative analyses of which have identified 194 compounds representing the major classifications of bioactive phytochemicals^37–40^. In seeking to further substantiate a central dopaminergic mechanism of action, we identified *l*-tetrahydropalmatine (*l*-THP) as a phytochemical constituent of interest. Its analgesic properties and potential therapeutic value in substance misuse are attributed to modulation of DA receptors^8,41^. Specifically, *l*-THP behaves functionally as a D1, D2 and D3 antagonist^8,42–45^ and moreover, exhibits the prototypical property of DA antagonists to increase DA release from axon terminals^46,47^. Consistent with DA autoreceptor modulation, low doses of *l*-THP (1–5 mg/kg) have been shown to enhance DA synthesis, release and metabolism, and most importantly, reverse the DA release-attenuating effects of the D2 agonist apomorphine^43,47^. To demonstrate that *l*-THP derived from Heantos-4 crosses the blood–brain barrier, a third experiment employed ultra high-pressure liquid chromatography/mass spectrometry (UHPLC/MS) methodology to detect *l*-THP in blood plasma and cerebrospinal fluid following oral administration of Heantos-4. A fourth experiment tested the hypothesis that *l*-THP may serve as an antagonist of the DA autoreceptor, capable of reversing the hypodopaminergic effects of quinpirole. Finally, a fifth experiment assessed whether *l*-THP as a key neuroactive ingredient in Heantos-4 could replicate the therapeutic effects of its parent formulation in morphine-withdrawn rats.

## Results

### Experiment 1: Effects of Heantos-4 in morphine-withdrawn rats

**Figure 1:**
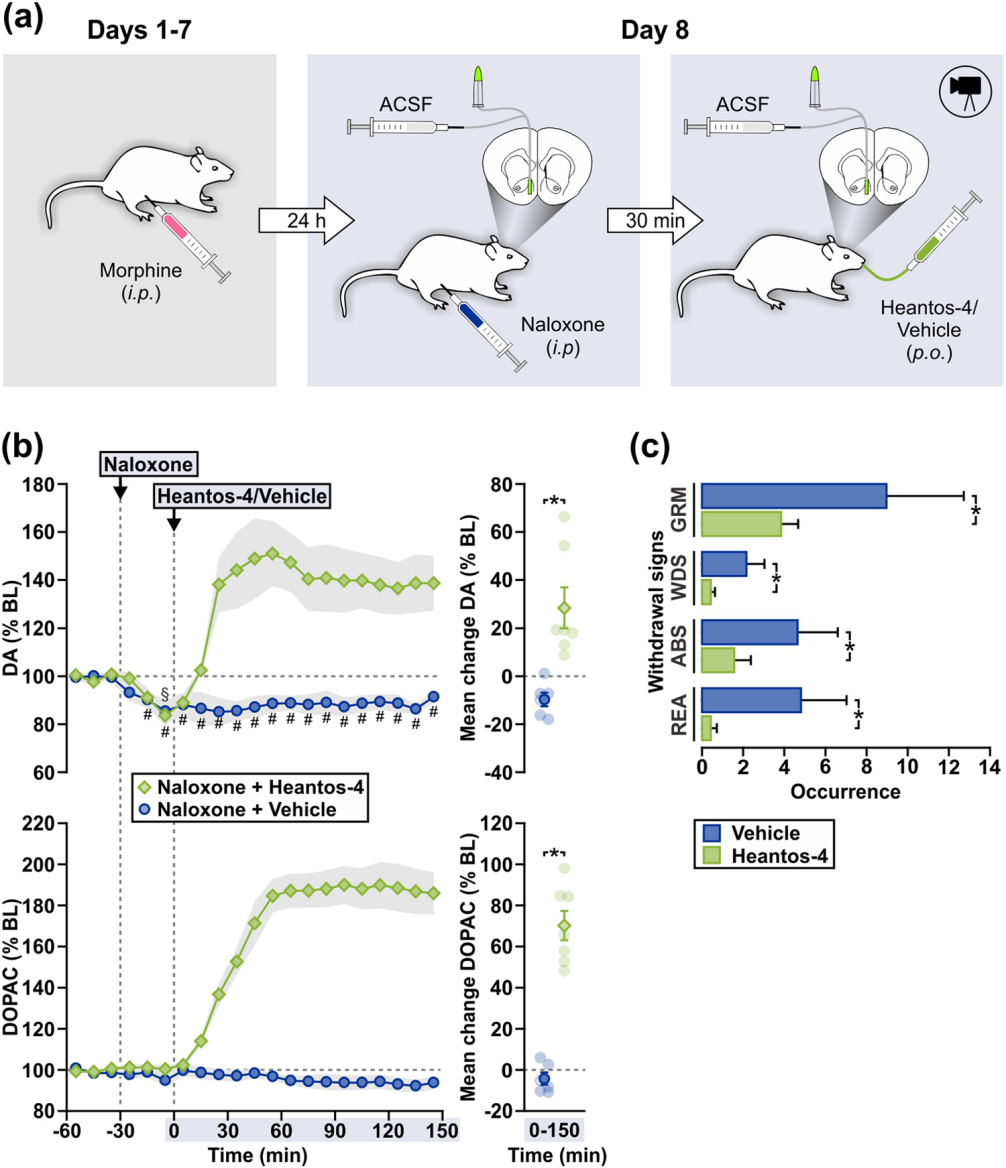
Heantos-4 stimulates DA efflux from a hypodopaminergic state and alleviates somatic withdrawal signs in morphine-dependent rats. (**a**) Schematic of treatments prior to (Days 1–7) and during microdialysis (Day 8) in Experiment 1. (**b**) In morphine-dependent rats, Heantos-4 (500 mg/kg, *p.o.*, n = 7; vehicle, n = 6) elicited an increase in DA and DOPAC efflux from a naloxone (10 mg/kg, *i.p.*)-induced state of hypodopaminergia in the NAc. Data (mean ± s.e.m) are presented as time-curves at 10 min intervals (line graphs) and as mean change during Time 0–150 min (individual data points also shown). Holm-Sidak multiple comparisons [^#(Vehicle), §(Heantos-4)^p < 0.05 vs. final sample prior to naloxone treatment; *p < 0.05]. (**c**) Reduction in naloxone-precipitated somatic withdrawal signs by Heantos-4 compared to vehicle, observed concurrently in rats undergoing microdialysis. The rate of occurrences (mean ± s.e.m.) are shown for face and body grooming (GRM), wet dog shakes (WDS), abdominal stretching (ABS) and rearing (REA) following Heantos-4 or vehicle treatment [Times 0–40, 60–80 and 120–140 min in (**b**)]. Two-sample Poisson rate test (*p < 0.05).

Rats were systemically injected with morphine (10 mg/kg/day, *i.p.*) on Days 1–7 days and naloxone (10 mg/kg, *i.p.*) on Day 8 (Fig. 1a). Naloxone evoked a significant reduction in DA efflux compared to baseline in all rats (Fig. 1b; vehicle-treated, Time − 40–150 min, F_18,90_ = 1.76, p = 0.04; Heantos-4-treated, Time − 40–0 min, F_3,18_ = 6.62, p < 0.01). In animals that subsequently received vehicle gavage, the hypodopaminergia persisted over a 3-h period. In contrast, administration of Heantos-4 (500 mg/kg, *p.o.*) 30 min *after* the onset of hypodopaminergia resulted an immediate increase in DA efflux that was sustained at values ~ 40% above pre-naloxone baseline levels for the duration of the experiment. Mean changes in DA (F_1,11_ = 15.91, p < 0.01) and DOPAC (F_1,11_ = 83.20, p < 0.01) efflux elicited by Heantos-4 were significantly higher than in the vehicle condition. The increase in dopaminergic activity following Heantos-4 was paralleled by a significant amelioration of naloxone-precipitated withdrawal signs (Fig. 1c). In comparison to vehicle, Heantos-4 treated rats showed a significantly lower occurrences of face and body grooming (estimate of difference = 5.14; 95% confidence interval 2.41–8.07; p < 0.01), wet dog shakes (estimate of difference = 1.74; 95% confidence interval 0.55–3.20; p = 0.01), abdominal stretching (estimate of difference = 3.10; 95% confidence interval 1.21–5.21; p = 0.049) and rearing (estimate of difference = 4.41; 95% confidence interval 2.72–6.44; p < 0.01).

### Experiment 2: Effect of Heantos-4 on quinpirole-induced hypodopaminergia

**Figure 2:**
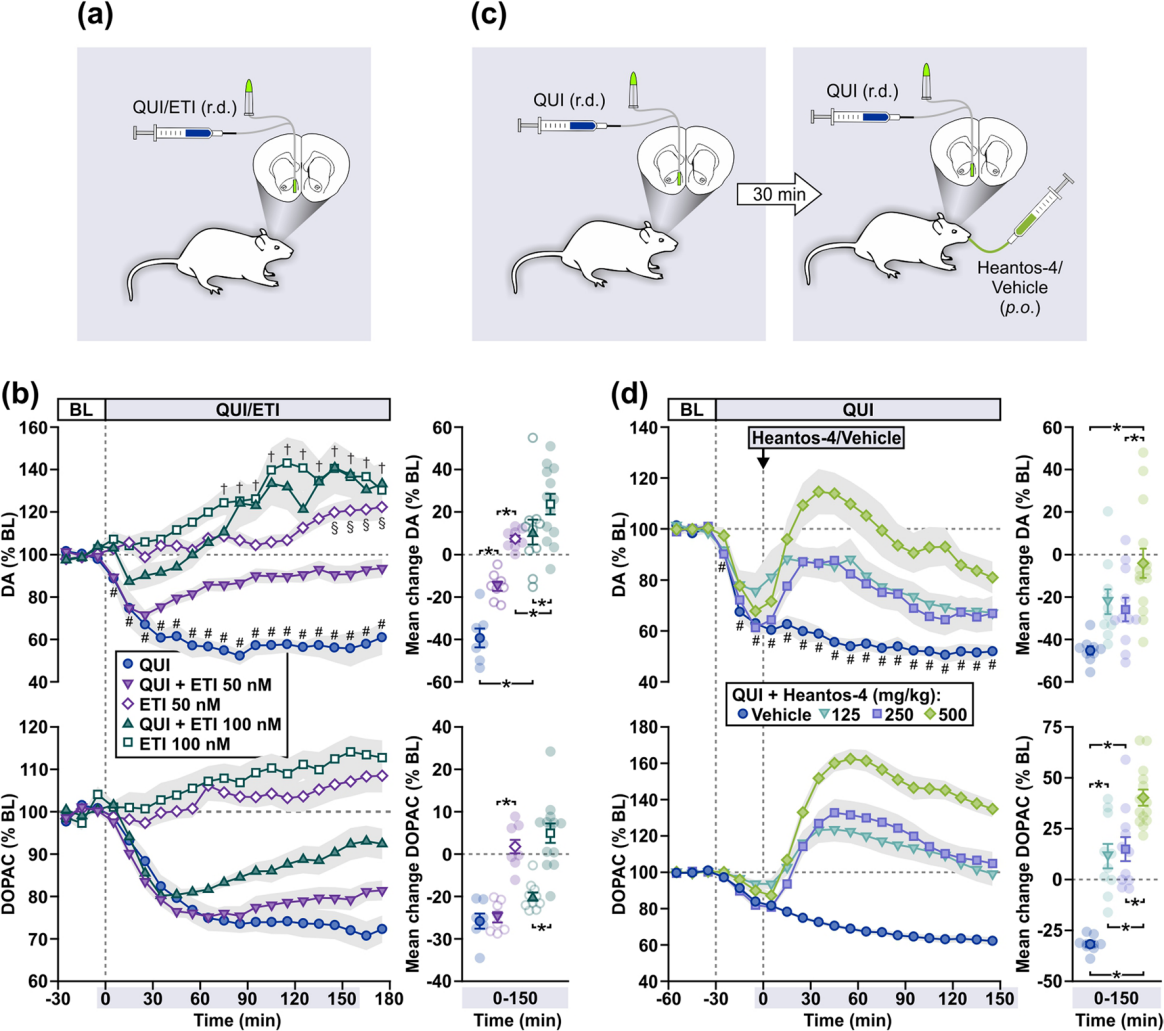
Heantos-4 reverses DA autoreceptor-mediated inhibition of DA efflux. (**a**,**c**) Schematics of treatments during microdialysis in Experiment 2. (**b**) Intra-NAc reverse-dialysis (r.d.) of quinpirole (QUI, 1 μM, n = 7) reduced while eticlopride (ETI, 50 and 100 nM, r.d.; n = 8 and 13, respectively) stimulated DA and DOPAC efflux. Co-application of QUI + ETI (1 μM + 50 nM and 1 μM + 100 nM, r.d.; n = 8 and 10, respectively) resulted in an ETI dose-dependent increase in DA and DOPAC efflux from an underlying hypodopaminergic state. (**d**) Heantos-4 (125, 250, 500 mg/kg and vehicle, *p.o.*; n = 14, 11, 10 and 9, respectively) dose-dependently reversed a QUI-induced reduction in DA and DOPAC efflux. Data (mean ± s.e.m) are presented as time-curves at 10 min intervals (line graphs) and as mean change during Time 0–150 min (individual data points also shown). Holm-Sidak multiple comparisons [^#(QUI), §(ETI 50), †(ETI 100)^p < 0.05 vs. final baseline (BL) sample; *p < 0.05].

We next assessed the presynaptic DA autoreceptor as a possible site at which Heantos-4 modulates DA release. First, we established an in vivo functional assay in which the DA autoreceptor could be selectively activated by appropriately low concentrations of the D2/D3 receptor agonist quinpirole (D2 Ki = 4.8 nM, D3 Ki = 24 nM)^48^ or inhibited by the D2 antagonist eticlopride (Ki =  ~ 0.92 nM)^49^ (Fig. 2a). In morphine-naïve rats, application of quinpirole (1 µM) directly into the NAc by reverse-dialysis was accompanied by a state of hypodopaminergia (Fig. 2b), with a significant reduction in basal DA levels reaching ~ 40% below baseline (F_18,108_ = 17.30, p < 0.01). Notably, quinpirole at 1 µM (or 0.26 ng/µL/min over 150 min) is within the lower range of the dose–response analyses of quinpirole (3–300 ng) previously shown to selectively affect DA autoreceptor function^50^. In contrast, eticlopride (50, 100 nM) dose-dependently elevated DA efflux above baseline (F_18,126_ = 3.37, p < 0.01 and F_18,126_ = 6.99, p < 0.01, respectively). Co-application of quinpirole and eticlopride resulted in an initial decline in DA efflux below baseline, which within 20–30 min returned to baseline values with the lower dose of eticlopride (50 nM) and were elevated above baseline with the higher dose of eticlopride (100 nM). As indicated by comparisons of mean changes in DA (F_4,41_ = 26.43, p < 0.01) and DOPAC (F_4,41_ = 27.36, p < 0.01) efflux, there was a significant dose-dependent effect of eticlopride to block quinpirole-induced hypodopaminergia.

The microdialysis-based DA autoreceptor assay was then used to assess the putative DA autoreceptor antagonist property of Heantos-4 in a separate group of rats (Fig. 2c). Intra-NAc reverse-dialysis of quinpirole (1 µM), followed 30 min later by vehicle gavage, resulted in a significant state of hypodopaminergia (F_18,144_ = 29.371, p < 0.01) (Fig. 2d). Subsequent administration of Heantos-4 (125, 250 and 500 mg/kg, *p.o.*) evoked an increase in DA efflux that quickly but transiently approached baseline levels, with the highest dose evoking increased efflux above pretreatment values. Statistical comparisons of mean changes in DA (F_3,40_ = 8.23, p < 0.01) and DOPAC (F_3,40_ = 37.69, p < 0.01) efflux indicated a significant dose-dependent reversal of quinpirole-induced hypodopaminergia by Heantos-4.

### Experiment 3: Detection of *l*-THP in blood plasma and cerebrospinal fluid following oral Heantos-4

**Figure 3:**
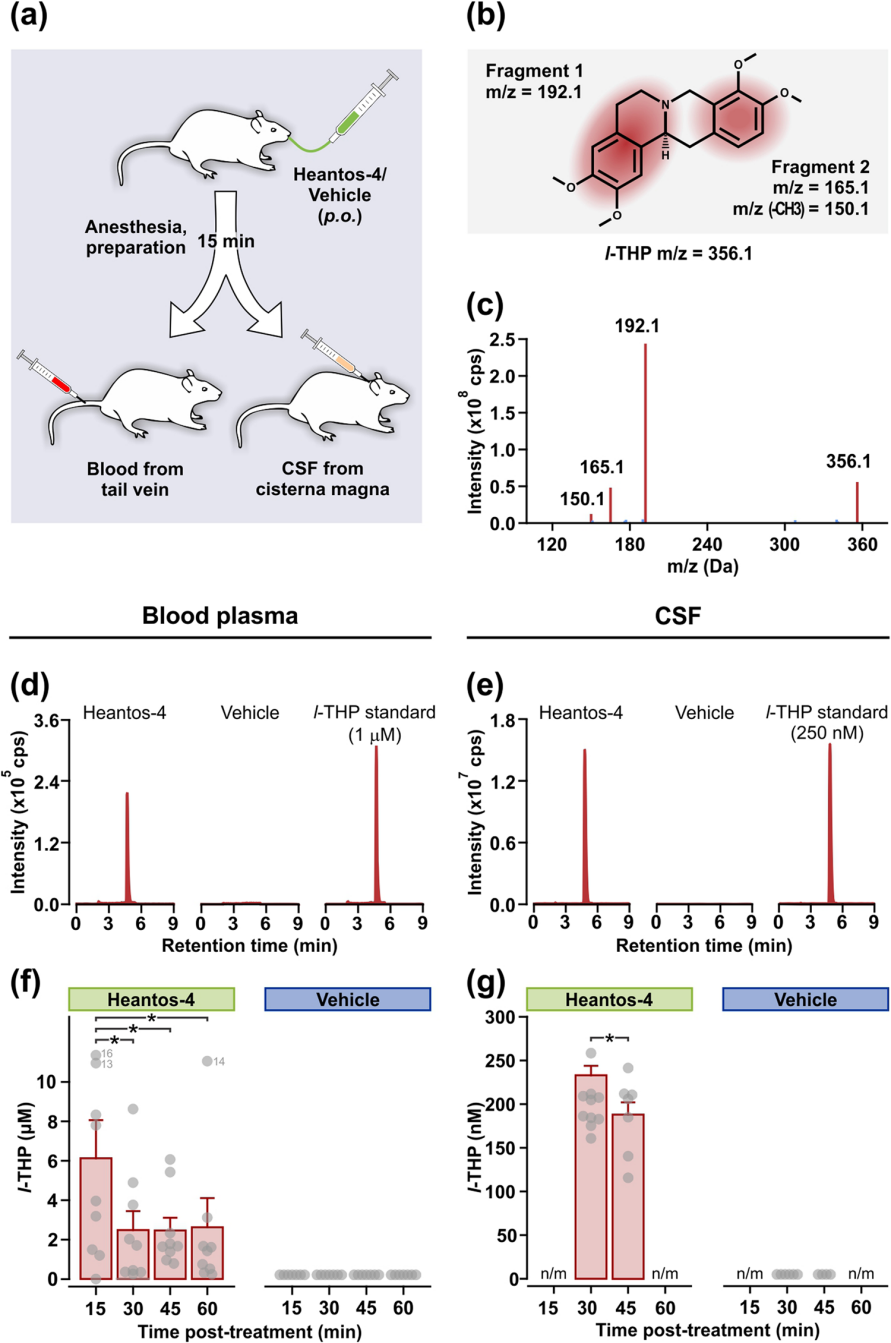
*l*-THP is detected in blood plasma and cerebrospinal (CSF) following oral Heantos-4. (**a**) Schematic of blood and CSF collection protocol following Heantos-4 treatment (500 mg/kg, *p.o.*) in Experiment 3. (**b**) Schematic representation of the unique fragmentation pattern of *l*-THP in positive ionization mode and associated m/z values. (**c**) Example of an UHPLC/MS/MS spectrum of an external *l-*THP standard (10 ng/mL) showing the m/z values associated with the expected fragments. (**d,e**) Representative ion chromatograms generated by UHPLC/MS/MRM to quantify *l*-THP content in tail vein blood plasma and cisterna magna CSF samples collected at 30 min post-Heanots-4, vehicle or no treatment (with addition of *l-*THP standard at 1 μM or 250 nM). (**f)** Concentration of *l-*THP detected in blood plasma following Heantos-4 (within-subject design, n = 9/time-point) or vehicle (within-subject design: n = 7/time-point). Holm-Sidak multiple comparisons (*p < 0.05). (**g**) Concentration of *l-*THP in CSF following Heantos-4 (between-groups design; 30 min, n = 10; 45 min, n = 7) or vehicle (between groups design; 30 min, n = 6; 45 min, n = 4). Two-tailed t-test (t = 2.475, df = 15, *p = 0.026). Data are shown as bar graphs (mean ± s.e.m) and individual data points. n.m., not measured.

Following Heantos-4 (500 mg/kg, *p.o.*) administration to rats, plasma from tail vein blood and cerebrospinal fluid from the cisterna magna were collected and analyzed for *l*-THP content using UHPLC/MS (Fig. 3a). As *l*-THP shares a similar molecular weight and tetrahydroprotoberberine chemical scaffold with numerous compounds in Heantos-4 (including those in Table 1), the UHPLC/MS method was optimized to detect the unique fragmentation pattern of *l*-THP molecules (Fig. 3b,c). This permitted the selective detection and quantification of *l*-THP in blood plasma and cerebrospinal fluid samples (Fig. 3d,e). At each of the collection times (15, 30, 45, 60 min post-treatment), *l*-THP levels were clearly present in plasma and cerebrospinal fluid of Heantos-4-treated rats; in contrast, *l*-THP was not detected in any of the samples from vehicle-treated rats (Fig. 3f,g). Given the obvious group differences (and zero variance in the vehicle-treated control group), between-group statistical analyses were not conducted. *l*-THP was detectable in plasma and cerebrospinal fluid up to 60 min post-Heantos-4 administration, establishing a clear main effect of time in the Heantos-4-treated group in blood plasma (F_3,24_ = 4.17, p = 0.02) and cerebrospinal fluid (2-tailed t-test, t = 2.48, df = 15, *p = 0.03). The concentration of *l*-THP were two orders of magnitude lower in the cerebrospinal fluid than in blood; nevertheless, the oral bioavailability of Heantos-4 as indexed by *l*-THP levels in cerebrospinal fluid (which ranged from 188 to 234 nM) were within the concentration range required for occupancy of D1 and D2 receptors (Ki = 124 ± 6 nM and 388 ± 78 nM, respectively) but not D3 (Ki = 1420 ± 220 nM) in the brain^42^.

**Table 1:** Neuropharmacological properties of tetrahydroprotoberberines identified to date in plant materials used in the preparation of Heantos-4^38^.

Tetrahydroprotoberberine	Therapeutic effect on opioid dependence	Dopamine receptor pharmacology	Mesolimbic dopamine activity	Molecular weight (g/mol)
*l*-Tetrahydropalmatine	Morphine, heroin, oxycodone^76–80^	D1 antagonist, D2 antagonist^8,42–45^	Increased DA release from axon terminals^46,47,66^	355.4
*l*-Stepholidine	Morphine, heroin^81–83^	D1 partial agonist, D1 antagonist, D2 antagonist^84–87^	Increased DA release from caudate slice^88^, increased DA neuron firing^89^	327.4
Corynoxidine	Unknown	Unknown	Unknown	371.4
(S)-Corydalmine	Morphine^90^	D2 antagonist^90^	Unknown	341.4
Thaicanine	Unknown	Unknown	Unknown	371.4

Molecular weights were obtained from PubChem Compound. Thaicanine represents both Thaicanine 4-O-β-d-glucoside and *l*-Thaicanine N-oxide.

### Experiment 4: Effect of *l*-THP on quinpirole-induced hypodopaminergia

**Figure 4:**
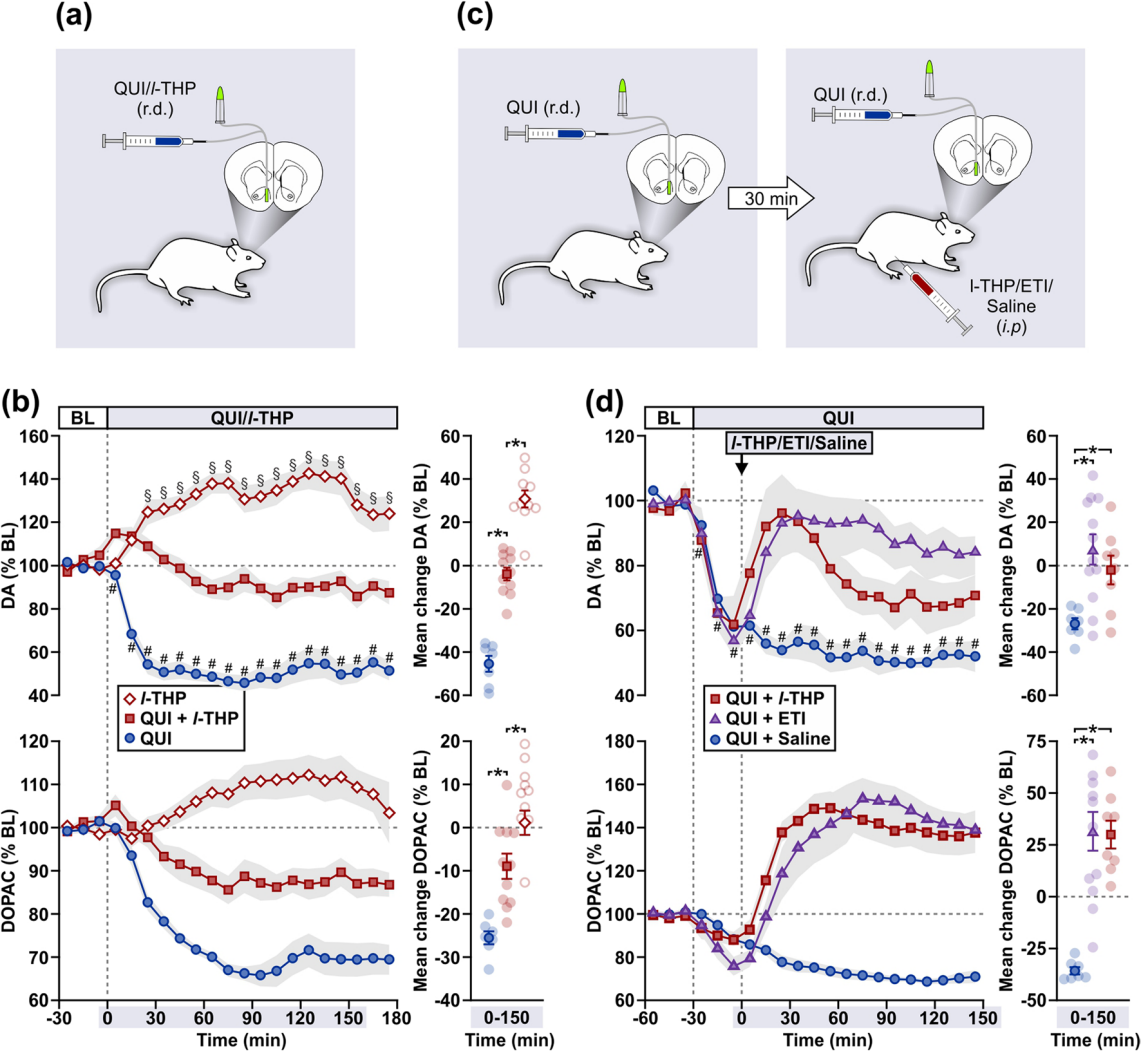
*l*-THP reverses DA autoreceptor-mediated inhibition of DA efflux. (**a**,**c**) Schematics of treatments during microdialysis in Experiment 4. (**b**) Intra-NAc reverse-dialysis (r.d.) of *l*-THP (50 μM, n = 10) increased DA and DOPAC efflux. Reduction of DA and DOPAC efflux by intra-NAc quinpirole (QUI, 1 μM, r.d.; n = 7) was mitigated by co-application of QUI + *l*-THP (1 μM + 50 μM, r.d.; n = 11). (**d**) Reduction of DA and DOPAC efflux by intra-NAc QUI (1 μM, r.d.) was subsequently reversed by systemic *l*-THP (5 mg/kg, *i.p.*, n = 8; saline, n = 7) or eticlopride (ETI, 0.3 mg/kg, *i.p.*, n = 12). Data (mean ± s.e.m) are presented as time-curves at 10 min intervals (line graphs) and as mean change during Time 0–150 min (individual data points also shown). Holm-Sidak multiple comparisons [^#(QUI), §(*l*-THP)^p < 0.05 vs. final baseline (BL) sample; *p < 0.05].

Given the evidence that *l*-THP derived from Heantos-4 crosses the blood–brain barrier, we next tested the hypothesis that *l*-THP, like its parent formulation, modulates DA efflux through antagonism of the DA autoreceptor. In our hands, intra-NAc reverse-dialysis of *l*-THP (50 µM) significantly increased DA efflux (F_18,162_ = 8.17, p < 0.01), whereas quinpirole (1 µM) induced a marked state of hypodopaminergia (F_18,108_ = 26.16, p < 0.01) (Fig. 4a,b). When quinpirole was co-infused with *l*-THP, DA efflux remained near pretreatment baseline levels. Importantly, mean changes in DA (F_2,25_ = 103.40, p < 0.01) and DOPAC (F_2,25_ = 31.15, p < 0.01) efflux were significantly higher in the quinpirole + *l*-THP group compared to quinpirole alone, suggesting that the quinpirole-induced hypodopaminergia was antagonized by the effects of *l*-THP in the NAc (i.e., via presynaptic DA autoreceptors on axon terminals as distinct from somatodendritic sites).

We then examined whether the effects of intra-NAc *l*-THP on the DA autoreceptor could be replicated by a systemic route of administration (Fig. 4c). Reverse-dialysis of quinpirole (1 µM) in the NAc, and followed by saline treatment (*i.p.*) 30 min later, resulted in a significant inhibition of DA efflux (F_18,108_ = 17.12, p < 0.01) (Fig. 4d). Subsequent treatment with *l*-THP (5 mg/kg, *i.p.*) evoked an initial increase in DA efflux that transiently restored baseline levels, and then gradually subsided to ~ 30% below baseline. Systemically administered eticlopride (0.3 mg/kg, *i.p.*), which served as an internal control for a DA autoreceptor antagonist, resulted in a rapid and sustained increase in DA efflux that approached baseline. As indicated by the mean change in DA (F_2,24_ = 6.98, p < 0.01) and DOPAC (F_2,24_ = 19.45, p < 0.01) efflux, dopaminergic activity was significantly higher in rats treated with either *l*-THP or eticlopride compared to saline.

### Experiment 5: Effects of *l*-THP in morphine-withdrawn rats

**Figure 5:**
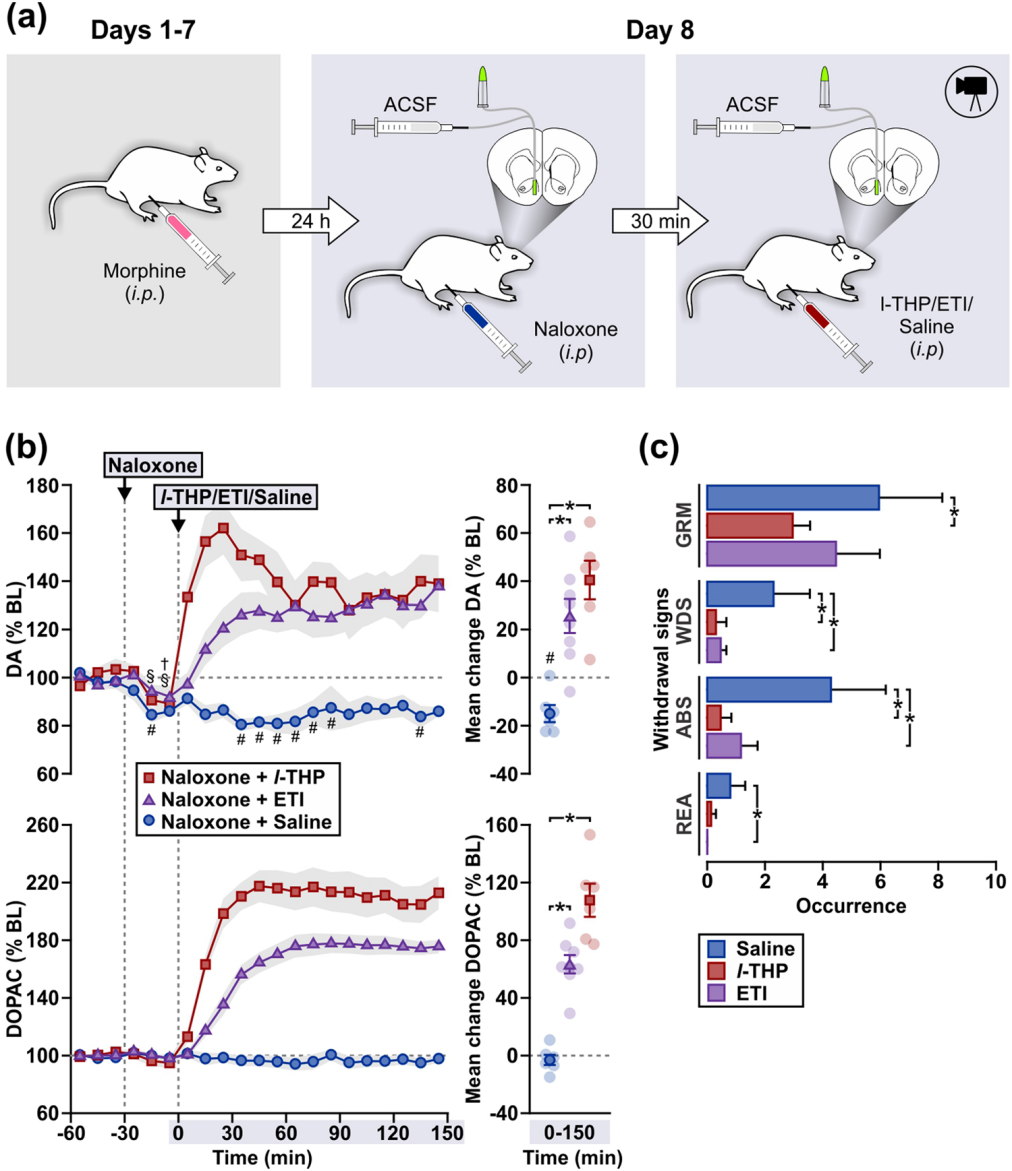
*l*-THP stimulates DA efflux from a hypodopaminergic state and alleviates somatic withdrawal signs in morphine-dependent rats. (**a**) Schematic of treatments prior to (Days 1–7) and during microdialysis (Day 8) in Experiment 5. (**b**) In morphine-dependent rats, systemic *l*-THP (5 mg/kg, *i.p.*, n = 6; saline, n = 6) and eticlopride (ETI, 0.3 mg/kg, i.p., n = 10) elicited an increase in DA and DOPAC efflux from a naloxone (10 mg/kg, *i.p.*)-induced state of hypodopaminergia in the NAc. Data (mean ± s.e.m) are presented as time-curves at 10 min intervals (line graphs) and as mean change during Time 0–150 min (individual data points also shown). Holm-Sidak multiple comparisons [^#(Saline), §(*l*-THP), †(ETI)^p < 0.05 vs. final sample prior to naloxone treatment; *p < 0.05]. (**c**) Reduction in naloxone-precipitated somatic withdrawal signs by *l*-THP and ETI compared to saline control, observed concurrently in rats undergoing microdialysis. Rate of occurrences (mean ± s.e.m.) for face and body grooming (GRM), wet dog shakes (WDS), abdominal stretching (ABS) and rearing (REA) following *l*-THP, ETI or saline treatment [Times 0–40, 60–80 and 120–140 min in (**b**)]. Two-sample Poisson rate test (*p < 0.05).

In a final experiment, we assessed whether *l*-THP alone could simulate therapeutic effects of Heantos-4 in morphine-withdrawn rats (Fig. 5a). Following seven days of morphine treatment (10 mg/kg, *i.p*.), naloxone (10 mg/kg, *i.p.*) evoked a significant reduction in DA efflux compared to pretreatment baseline in all rats (saline-treated, Time − 40–150 min, F_18,90_ = 1.98, p = 0.02; *l*-THP-treated, Time − 40–0 min, F_3,15_ = 5.70, p < 0.01; eticlopride-treated, Time − 40–0 min, F_3,21_ = 3.06, p = 0.05; Fig. 5b). In animals subsequently injected with saline, the hypodopaminergia persisted over a 3-h period, replicating the observations in Experiment 1. Subsequent treatment with either a single dose of *l*-THP (5 mg/kg, *i.p.*) or eticlopride (0.3 mg/kg, *i.p.*), the latter once again serving as an internal control for D2 antagonism, resulted in an immediate increase in DA efflux from a hypodopaminergic state. Both *l*-THP and eticlopride treatments evoked mean changes in DA (F_2,18_ = 17.66, p < 0.01) and DOPAC (F_2,18_ = 51.12, p < 0.01) that were significantly higher than in the vehicle condition. Importantly, , the moderate increases in DA efflux, induced by l-THP ranging from ~ 30 to 50% above baseline, were accompanied by a significant amelioration of naloxone-precipitated withdrawal signs (Fig. 5c). In comparison to saline, *l*-THP-treated rats showed a significantly lower occurrence of face and body grooming (estimate of difference = 3.00; 95% confidence interval 0.62–5.49; p = 0.02), wet dog shakes (estimate of difference = 2.00; 95% confidence interval 0.76–3.50; p < 0.01) and abdominal stretching (estimate of difference = 3.83; 95% confidence interval 2.17–5.78; p < 0.01). In comparison to saline, eticlopride-treated rats displayed significantly lower occurrences of wet dog shakes (estimate of difference = 1.83; 95% confidence interval 0.67–3.34; p < 0.01,) abdominal stretching (estimate of difference = 3.13; 95% confidence interval 1.47–5.13; p < 0.01) and rearing (estimate of difference = 0.83; 95% confidence interval 0.26–1.83; p = 0.01).

### Basal DA levels in morphine-treated and -naïve rats (Experiments 1–5)

D2 receptors are vulnerable to regulatory changes in response to withdrawal from repeated morphine treatments^51^. To assess whether DA autoreceptor-mediated feedback inhibition was altered by withdrawal from morphine treatment, we compared basal DA concentrations in morphine-dependent and –naïve rats. On microdialysis day, DA concentrations (mean ± s.e.m.) of samples collected during the baseline period prior to naloxone treatment in rats that received the 7th morphine injection 24 h earlier (Experiments 1 and 5, n = 33) and control rats naïve to morphine (Experiments 2 and 4, n = 145) were 1.05 ± 0.08 nM and 1.09 ± 0.05 nM, respectively. Analysis of the basal DA values observed in each of the experiments (Supplementary Table S1) revealed no statistical difference [H_5_ = 9.16, p = 0.10].

## Discussion

Current non-opioid detoxification therapies target the dysfunctional states of the dopaminergic and noradrenergic systems which are associated with affective and somatic correlates of opioid withdrawal^18,52^. For example, in morphine-dependent animals, clonidine reverses a hypodopaminergic condition^18^ and noradrenergic hyperactivity as well as activating α2-adrenergic and imidazoline-1 receptors^53,54^. However, while the combined agonism of both receptor types synergistically prevents inhibition of midbrain DA neuron firing induced by naltrexone^54^, imidazoline-1 receptor activity is also associated with hypotensive side-effects that limit utility^55^.

The present findings indicate that a modest upregulation of mesolimbic DA activity, and a consequent reversal of a hypodopaminergic state in the NAc, underlies the ability of Heantos-4 to attenuate somatic signs of withdrawal in morphine-dependent rats. The effectiveness of a single treatment with Heantos-4 was notable, as was its immediacy (on the order of minutes) in either preventing the appearance^19^ or mitigating the hypodopaminergic and somatic features of withdrawal after their onset (present study).

The identification of *l*-THP as a key active ingredient, which crosses the blood–brain barrier (Fig. 3) and shows a therapeutic potency equal to its parent formulation in morphine-dependent rats (Figs. 1 and 5) further substantiates a central dopaminergic mechanism of action. In line with the known characterization of *l*-THP as a D2/3 antagonist^8,42–45^, application of a low dose directly in the NAc reverses hypodopaminergia induced by the D2/3 agonist quinpirole (Fig. 4) as well as the D2 agonist apomorphine^43,47^, consistent with the selective antagonism of DA autoreceptors in the NAc. Findings in mice with D2 receptor mutations suggest that the D2S isoform in particular may play an important role in opioid dependence^28^. Specifically, the capacity of D2S to presynaptically assert auto-inhibition on DA activity appears critical to dampening supra-physiological increases in DA release evoked by opioids; conversely, it is plausible that blockade of D2S autoreceptors could normalize basal DA activity from a hypoactive state. Based on these premises, we propose that the anti-hypodopaminergic effects of *l*-THP reflect a putative antagonist action on presynaptic DA autoreceptors, which could serve as a novel therapeutic strategy to ‘normalize’ dysfunction of the mesolimbic DA system in OUD.

Importantly, enhancement of DA function in the NAc, either by drugs or endogenous opioids, is effective in the suppression of tonic pain^56^, as alleviation of pain contributes to successful detoxification from opioids^2^. Accordingly, the analgesic properties of *l*-THP^8^, mediated in part by elevation of DA release in the NAc may contribute to management of withdrawal-associated pain. It should also be noted that effects of *l*-THP (administered systemically) on somatic signs of withdrawal are unlikely to be mediated solely by the NAc; indeed, it will be important to determine the involvement of somatodendritic DA autoreceptors in regulating midbrain DA cell activity.

Although our data suggest that the anti-hypodopaminergic effects of *l*-THP and Heantos-4 involve antagonism of the DA autoreceptor, other mechanisms, such as DA transporter (DAT) inhibition, could also contribute to this effect. It is notable that different DAT inhibitors are distinguishable by the DOPAC responses they evoke: both cocaine and *d*-amphetamine give rise to an increase in extracellular DA accompanied by a decrease in DOPAC levels^57–59^ whereas methylphenidate elicits increases in DA levels, with relatively little to no change in DOPAC^60,61^. In this regard, the effects of Heantos-4 and *l*-THP to consistently enhance extracellular levels of DOPAC do not reflect patterns seen following disruption of DAT function. In fact, the robust and sustained elevation of both DA and DOPAC efflux (Figs. 1, 2, 4 and 5) resembles the upregulation of DA activity observed with D2 antagonists^62,63^.

The comparable potency of Heantos-4 and *l*-THP to reverse hypodopaminergia induced by either quinpirole in drug-naïve or naloxone in morphine-dependent rats, while also ameliorating key features of opioid-withdrawal, is compelling. Indeed, *l*-THP has been explored as a treatment option for heroin and cocaine-use disorders, with the caveat that doses higher than the recommended therapeutic range for humans (60–180 mg) are associated with adverse side-effects, such as sedation and anhedonia^41,42,78^. Higher, sedation-inducing doses of *l*-THP may involve antagonism of postsynaptic D2 receptors as well as a non-dopaminergic (benzodiazepine-like) mechanisms via positive allosteric modulation of GABA-A receptors^64^. It is also noteworthy that non-sedative doses of *l*-THP (below 10 mg/kg in rodents)^65–69,77^, administered in combination with a low dose of a µ-opioid receptor antagonist naltrexone is more effective than *l*-THP alone in blocking cocaine-seeking by rats^70^. While the precise quantity of *l*-THP in the Heantos-4 formulation in relation to the threshold for inducing side-effects remains to be determined, our behavioral observation of Heantos-4-treated rats indicate that *l*-THP within the Heantos-4 formulation is well-tolerated, with no adverse side-effects aside from a mild state of somnolence (present study and ref.^19^).

The use of combined botanical extracts with a range of pharmacological effects is a prominent feature of many botanical formulations^71^, and may account for the clinical success of Heantos-4 to mitigate opioid withdrawal. While the present study focused on *l*-THP as a mediator of therapeutic effects of Heantos-4, this formulation contains other phytochemicals in the tetrahydroprotoberberine family with known effects on brain dopaminergic activity and opioid dependence (Table 1). Further investigation of these and other botanicals within the Heantos-4 formulation, with a focus on interactions between the dopaminergic and noradrenergic systems^52^ as well as on both somatic and affective features of opioid withdrawal^2,13^ are warranted in order to gain a fuller understanding of factors that may explain the promising clinical effects of Heantos-4 in facilitating opioid detoxification. Furthermore, we recognize that the present emphasis on somatic signs of withdrawal from opioid dependence represents only one of several important features of OUD. In particular, the relationship between hypodopaminergia and the dysphoric effects of opioid withdrawal merit further study^13^, and experiments are underway to extend our earlier preclinical assessments of affective measures of opioid withdrawal (e.g., naloxone-precipitated conditioned place avoidance; see ref.^19^). Future studies utilizing more sophisticated models of addiction, including self-administration paradigms, will explore the potential effectiveness of acute and repeated Heantos-4 treatments on opioid craving, seeking and relapse.

In summary, the present preclinical insights into the neuropharmacological, neurochemical and behavioral effects of Heantos-4 in morphine-dependent rats complement the promising clinical observations in Vietnam and provide a compelling rationale for further investigations of this non-opioid based formulation as a gateway treatment for OUD.

## Methods

### Subjects and surgery

All experimental procedures were conducted in accordance with the ethical standards set by the Canadian Council on Animal Care and approved by the University of British Columbia Animal Care Committee.

Male Sprague–Dawley rats (250–275 g) from Charles River (St. Constant, Canada) were pair-housed upon arrival in a colony room that was maintained at ~ 21 °C with a 12-h light/dark cycle (lights on at 7 p.m.).

Rats in microdialysis experiments were implanted with bilateral guide cannulae directly over the NAc (from bregma, + 1.7 mm anterio-posterior, ± 1.1 mm mediolateral; from dura, − 1.0 mm dorso-ventral) as previously described^19^. During recovery (7 days minimum) rats were individually housed, with food and water available *ad libitum*.

### In vivo microdialysis and HPLC

Microdialysis probes were concentric in design and assembled in-house as previously described^19^. Prior to implantation, probes were connected to an Instech liquid swivel (Plymouth Meeting, PA, US) and thoroughly flushed with the perfusion medium, artificial cerebrospinal fluid which contained (in mM): sodium phosphate buffer (10), CaCl_2_ (1.2), KCl (3), MgCl_2_ (1) and NaCl (147) at pH 7.4. The probes were then inserted via the guide cannulae into the NAc (2 mm membrane length spanned − 4.8 to − 6.8 mm below dura) without the use of anesthesia. Rats remained in a plexiglass chamber (40 × 40 × 40 cm) with food and water overnight. Probes were continuously perfused with artificial cerebrospinal fluid at 1 µL/min. Sample collection began 16–18 h post-implantation at 10 min intervals. Once DA levels stabilized (with less than 10% fluctuation over four consecutive samples), each rat received its predesignated treatment and sampling continued for several hours.

Microdialysis samples were analyzed immediately upon collection using reverse-phase HPLC with electrochemical detection as previously described^19^. The identification and quantification of DA and dihydroxyphenylacetic acid (DOPAC) content in microdialysis samples were based on a 3-point calibration of external standards prepared in artificial cerebrospinal fluid.

The placement of microdialysis probes in the brain was verified using histological methods as previously described^19^. As shown in Supplementary Fig. S1, tracts formed by the membrane portion of microdialysis probe were localized to the shell/core border of the NAc, spanning 1.2 to 2.2 mm anterior to bregma.

### Drugs and preparation

Heantos-4, produced by the Sung lab, was received as a dry granular powder that was not readily soluble in water. Therefore, the drug was prepared daily as a suspension in 0.5% (w/v) carboxymethyl cellulose in H_2_O (vehicle) and administered 2.0–2.5 mL per os (*p.o.*) using a soft rubber feeding tube. The dose-range (125, 250 and 500 mg/kg) and the oral route of administration were based on the clinical treatment prescribed for opioid detoxification in Vietnam^11^.

Quinpirole hydrochloride, eticlopride hydrochloride and naloxone hydrochloride were obtained from Sigma-Aldrich (Oakville, Canada). Morphine sulfate and *l*-THP were obtained from Unipharm Wholesale Drugs Limited (Richmond, Canada) and Santa Cruz Biotechnology (Dallas, Texas, US), respectively. quinpirole, eticlopride and *l-*THP were prepared daily in 0.5% (v/v) dimethyl sulfoxide in artificial cerebrospinal fluid for application by intra-NAc reverse-dialysis. Morphine and naloxone were prepared daily in sterile saline for intraperitoneal (*i.p.*) injections. Eticlopride and *l-*THP were initially dissolved in ~ 100 µL 0.1 M H_2_SO_4_ and brought to final volume with saline (pH was adjusted to 5.5 using NaOH). All *i.p.* injections were administered at 1 mL/kg.

### Concurrent microdialysis and assessment of withdrawal in morphine-dependent rats (Experiments 1 and 5)

The rat model of morphine dependence employed here is modified from a previously described protocol^18^. One week following surgery, morphine (10 mg/kg, *i.p.*) was administered on Days 1–7. Approximately 5 h after the final injection, rats were implanted with microdialysis probes in the NAc (left and right hemispheres counterbalanced). The following morning (on Day 8), with stable baseline DA levels established, the µ-opioid receptor antagonist naloxone (10 mg/kg, *i.p.*) was administered, followed 30 min later by either Heantos-4 (500 mg/kg, *p.o.*), vehicle (*p.o.*), *l*-THP (5 mg/kg, *i.p.*) or saline (*i.p.*).

Concurrent with microdialysis sampling, rats were assessed for naloxone-precipitated withdrawal signs at 0–20 min, 60–80 min and 120–140 min following administration of Heantos-4, *l*-THP, eticlopride, or control treatment (saline or vehicle). Using real-time monitoring and video recordings, a researcher blind to treatment conditions recorded the occurrences of face and body grooming, wet dog shakes, abdominal stretching and rearing.

### Microdialysis/reverse-dialysis experiments in morphine-naïve rats (Experiments 2 and 4)

Rats with probes in the NAc were randomly assigned to one of the following treatment condition: intra-NAc reverse-dialysis of quinpirole (1 µM), eticlopride (50 or 100 nM) or quinpirole (1 µM) + eticlopride (50 or 100 nM); intra-NAc reverse-dialysis of quinpirole (1 µM) followed 30 min later by Heantos-4 (125, 250 or 500 mg/kg, *p.o.*) or vehicle; intra-NAc reverse-dialysis of quinpirole (1 µM), *l-*THP (50 µM) or quinpirole (1 µM) + *l*-THP (50 µM); and intra-NAc reverse-dialysis of quinpirole (1 µM) followed 30 min later by eticlopride (0.3 mg/kg, *i.p.*), *l-*THP (5 mg/kg, *i.p.*) or saline. Following a minimum of 7 days of rest, rats were implanted with probes in the NAc of the opposite hemisphere for a second microdialysis session and administered a different drug treatment.

### Collection and UHPLC/MS analysis of blood plasma and cerebrospinal fluid samples (Experiment 3)

#### Blood collection

Following Heantos-4 (500 mg/kg, *p.o.*) or vehicle administration, anesthesia was induced with 4% isoflurane gas mixed with oxygen and maintained with 2.0–2.5% isoflurane for the remainder of the procedure. Blood from the lateral tail vein was collected using a previously described procedure^72^. Samples were collected serially at 15, 30, 45 and 60 min post-treatment (~ 1 mL /time point) from each rat (i.e., within-subject design) and deposited into Eppendorf tubes coated with ethylenediaminetetraacetic acid (0.1 M). To isolate plasma, blood samples were centrifuged at 4 ℃ for 10 min at 1300×*g* and then filtered through an ultrafiltration cartridge (30 kDa cut-off) to remove proteins for UHPLC/MS analysis.

#### Cerebrospinal fluid collection

Following oral gavage of Heantos-4 (500 mg/kg, *p.o.*) or vehicle, rats were anesthetized with urethane (25 g/7 mL) and, in a prone position, the head was secured at a downward (~ 45°) angle from horizontal. The dissection of tissue to reveal the cisterna magna was performed according to a previously described procedure^73^. A 28 G ½ in. needle attached to a 1 mL syringe was inserted through the dural surface of the cisterna magna at a 30° angle. The cerebrospinal fluid was carefully aspirated into the syringe until ~ 100 µL of fluid was collected, deposited into an Eppendorf tube and stored at − 80 ℃. In comparison to blood, the volume of cerebrospinal fluid is significantly smaller, and permits collection of a single sample per time-point. Thus, a between-group design was employed to collect cerebrospinal fluid samples at 30 and 45 min post-gavage. Time-constraints related to experimental procedures (e.g., oral administration, induction of anesthesia and tissue dissection prior to cerebrospinal fluid collection) precluded sample collection at 15 min.

#### UHPLC/MS system

Analysis of blood plasma and cerebrospinal fluid samples were conducted using an UHPLC/MS system consisting of an Agilent 1290 Infinity Binary Pump, Sampler, Thermostat, and Thermostatted Column Compartment (Mississauga, Canada) connected to an AB SCIEX QTRAP 5500 hybrid linear ion trap triple quadrupole mass spectrometer equipped with a Turbo Spray source (Concord, Canada). The mass spectrometer was operated in positive ionization mode, and data were acquired using the Analyst 1.5.2. software on a Microsoft Windows XP Professional operating platform.

A Waters Acquity UHPLC BEH C18 column (1.7 µm particle, 2.1 × 100 mm; Mississauga, Canada) was used for chromatographic analysis. The mobile phase was composed of 0.1% formic acid in deionized water (Solvent A) and 0.1% formic acid in methanol (Solvent B). The flow rate was 200 µL/min with 15% solvent B as initial condition (t = 0 min), increasing to 60% solvent B to t = 4 min, then increasing to 85% solvent B to t = 6 min, then held for 1.5 min until t = 7.5 min. The gradient was then reverted back to original conditions of 15% solvent B from t = 7.6 min and stabilized for 1.5 min before the next injection. The total run time was 9 min. The injection volume was 10 µL.

The mass spectrometer was operated with electrospray ionization (ESI) in multiple reaction monitoring (MRM) mode with the following parameters: ionization voltage (4500 V), source temperature (450 °C), curtain gas (30 units), ion source gas 1 (40 units), ion source gas 2 (60 units), and collision gas was set to high. Nitrogen was used for all gases. Both Q1 and Q3 quadrupoles were at unit mass resolution, entrance potential was 10 and dwell time was 150 ms.

#### Identification and quantification of l-THP

The following method was developed to identify *l-*THP, as distinct from other tetrahydroprotoberberines and related small molecules with similar molecular weights (Table 1). A UHPLC/MS/MS full scan was performed using direct infusion of *l-*THP (10 ng/mL) into the mass spectrometer, which generated a mass spectra consistent with the known fragmentation pattern of *l-*THP molecules^74^. The m/z values of the three main fragments (150, 165 and 192) were then used to set the MRM transition parameters for quantification of *l-*THP content in biological samples (Fig. 4b): *l-*THP m/z 356 → 150 (declustering potential (DP) 131, collision energy (CE) 35, collision cell exit potential (CXP) 15), m/z 356 → 165 (DP 131, CE 33, CXP 14) and m/z 356 → 192 (DP 131, CE 35, CXP 20); internal standard m/z 268 → 237 (DP 66, CE 23, CXP 6).

Quantification of *l-*THP levels in plasma samples was based on a 5-point external calibration of *l-*THP. The standards were prepared in blood plasma sampled from normal untreated rats, processed in the same manner as experimental samples (i.e., ultra-filtration) and analyzed under the UHPLC/MS/MRM conditions described above. Quantification of *l-*THP levels in cerebrospinal fluid samples were based on a 6-point external calibration of *l-*THP standards prepared in cerebrospinal fluid from normal untreated rats and analyzed using the same UHPLC/MS/MRM conditions.

### Data presentation and statistical analyses

#### Microdialysis data

DA and DOPAC data for each subject were normalized to baseline values, with 100% representing mean DA or DOPAC content in the three samples prior to treatment. For statistical purposes, the mean value during Time 0–150 min was calculated for each subject. Between-group comparisons were conducted using a one-way analysis of variance (ANOVA). Within-group comparisons against baseline (mean of values during Time − 30–0 min) were conducted using a one-way repeated measures (RM) ANOVA. These were followed when appropriate by the Holm-Sidak method of multiple comparisons (overall significance level was p < 0.05). Basal DA levels were determined as the mean of content of DA during the baseline period (final  30 min), and the Kruskal–Wallis one-way ANOVA on ranks test was used to determine between-group differences.

#### l-THP levels in plasma and cerebrospinal fluid

Analysis of plasma levels of *l-*THP involved a one-way RM ANOVA followed by Holm-Sidak method of multiple comparisons (overall significance level was p < 0.05). Statistical analyses of cerebrospinal fluid levels of *l-*THP involved a two-tailed t-test.

#### Somatic withdrawal signs

Between-group comparisons of the incidence of individual withdrawal signs were conducted using a two-sample Poisson ratio test (two-tailed, 95% CL).

The ‘box plot’ method of identifying outliers was applied to all data sets^75^. SigmaPlot (version 12.3) and SigmaXL (version 9) was used to perform the statistical analyses.

## Supplementary information

Supplementary Information.

## Data Availability

All data generated or analysed during this study are included in this published article (and its Supplementary Information files).

## References

[1] United Nations Office on Drugs and Crime. *World drug report 2019* (United Nations, New York, 2019). https://wdr.unodc.org/wdr2019/.

[2] Kosten TR, Baxter LE (2019). Effective management of opioid withdrawal symptoms: A gateway to opioid dependence treatment. Am. J. Addict..

[3] Pergolizzi JV, Raffa RB, Rosenblatt MH (2020). Opioid withdrawal symptoms, a consequence of chronic opioid use and opioid use disorder: Current understanding and approaches to management. J. Clin. Pharm. Ther..

[4] Phillips AG, Krausz MR (2018). Utilizing resources of neuropsychopharmacology to address the opioid overdose crisis. Neuropsychopharmacol. Rep..

[5] U.S. Food and Drug Administration. *FDA approves the first non-opioid treatment for managment of opioid withdrawal symptoms in adults* [Press Release] (2018, May 16). https://www.fda.gov/news-events/press-announcements/fda-approves-first-non-opioid-treatment-management-opioid-withdrawal-symptoms-adults.

[6] Kuszmaul AK, Palmer EC, Frederick EK (2020). Lofexidine versus clonidine for mitigation of opioid withdrawal symptoms: A systematic review. J. Am. Pharm. Assoc..

[7] Lu L (2009). Traditional medicine in the treatment of drug addiction. Am. J. Drug Alcohol Abuse.

[8] Chu H, Jin G, Friedman E, Zhen X (2008). Recent development in studies of tetrahydroprotoberberines: Mechanism in antinociception and drug addiction. Cell. Mol. Neurobiol..

[9] Shi J (2006). Traditional Chinese medicine in treatment of opiate addiction. Acta Pharmacol. Sin..

[10] Aldhous P (2005). Drug rehabilitation: Cold turkey, Vietnamese style. Nature.

[11] Sung, T. V., Chinh, N. B., Dan, T. K. & Quang, N. About the anti-drug medication Heantos-4. *Asia Pac. Psychiatry* **7,** abstr. 117 (2015).

[12] Melis M, Spiga S, Diana M (2005). The dopamine hypothesis of drug addiction: Hypodopaminergic state. Int. Rev. Neurobiol..

[13] Koob GF (2020). Neurobiology of opioid addiction: Opponent process, hyperkatifeia, and negative reinforcement. Biol. Psychiatry.

[14] Mazei-Robison MS, Nestler EJ (2012). Opiate-induced molecular and cellular plasticity of ventral tegmental area and locus coeruleus catecholamine neurons. Cold Sping Harb. Perspect. Med..

[15] Kosten TR, George TP (2002). The neurobiology of opioid dependence: Implications for treatment. Sci. Pract. Perspect..

[16] Diana M (2011). The dopamine hypothesis of drug addiction and its potential therapeutic value. Front. Psychiatry.

[17] Martinez D (2012). Deficits in dopamine D(2) receptors and presynaptic dopamine in heroin dependence: Commonalities and differences with other types of addiction. Biol. Psychiatry.

[18] Pothos E, Rada P, Mark GP, Hoebel BG (1991). Dopamine microdialysis in the nucleus accumbens during acute and chronic morphine, naloxone-precipitated withdrawal and clonidine treatment. Brain Res..

[19] Dias C, Ahn S, Ma B, Sung TV, Phillips AG (2016). Behavioural and neurochemical assessment of Heantos 4 on preclinical models of morphine-dependence. J. Addict. Res. Ther..

[20] Rossetti ZL, Hmaidan Y, Gessa GL (1992). Marked inhibition of mesolimbic dopamine release: A common feature of ethanol, morphine, cocaine and amphetamine abstinence in rats. Eur. J. Pharmacol..

[21] Acquas E, Di Chiara G (1992). Depression of mesolimbic dopamine transmission and sensitization to morphine during opiate abstinence. J. Neurochem..

[22] Crippens D, Robinson TE (1994). Withdrawal from morphine or amphetamine: Different effects on dopamine in the ventral-medial striatum studied with microdialysis. Brain Res..

[23] Diana M, Pistis M, Muntoni A, Gessa G (1995). Profound decrease of mesolimbic dopaminergic neuronal activity in morphine withdrawn rats. J. Pharmacol. Exp. Ther..

[24] Diana M, Muntoni AL, Pistis M, Melis M, Gessa GL (1999). Lasting reduction in mesolimbic dopamine neuronal activity after morphine withdrawal. Eur. J. Neurosci..

[25] Volkow ND, Wise RA, Baler R (2017). The dopamine motive system: Implications for drug and food addiction. Nat. Rev. Neurosci..

[26] Harris GC, Aston-Jones G (1994). Involvement of D2 dopamine receptors in the nucleus accumbens in the opiate withdrawal syndrome. Nature.

[27] Laviolette SR, Nader K, van der Kooy D (2002). Motivational state determines the functional role of the mesolimbic dopamine system in the mediation of opiate reward processes. Behav. Brain Res..

[28] Rouge-Pont F (2002). Changes in extracellular dopamine induced by morphine and cocaine: Crucial control by D2 receptors. J. Neurosci..

[29] Sokoloff P, Giros B, Martres MP, Bouthenet ML, Schwartz JC (1990). Molecular cloning and characterization of a novel dopamine receptor (D3) as a target for neuroleptics. Nature.

[30] Radl D (2018). Differential regulation of striatal motor behavior and related cellular responses by dopamine D2L and D2S isoforms. Proc. Natl. Acad. Sci. USA.

[31] Meller E, Bohmaker K, Goldstein M, Basham DA (1993). Evidence that striatal synthesis-inhibiting autoreceptors are dopamine D3 receptors. Eur. J. Pharmacol..

[32] Ford CP (2014). The role of D2-autoreceptors in regulating dopamine neuron activity and transmission. Neuroscience.

[33] Sarre S, Vandeneede D, Ebinger G, Michotte Y (1998). Biotransformation of L-DOPA to dopamine in the substantia nigra of freely moving rats: Effect of dopamine receptor agonists and antagonists. J. Neurochem..

[34] Sharp T, Zetterstrom T, Collin AK, Ungerstedt U (1987). The D-2 agonist quinpirole releases striatal dopamine in vivo. Eur. J. Pharmacol..

[35] Imperato A, Di Chiara G (1988). Effects of locally applied D-1 and D-2 receptor agonists and antagonists studied with brain dialysis. Eur. J. Pharmacol..

[36] Kennedy RT, Jones SR, Wightman RM (1992). Dynamic observation of dopamine autoreceptor effects in rat striatal slices. J. Neurochem..

[37] Anh NTH (2013). Phytochemical study on the plants of the antidrug medication Heantos 4. Part 3. Homoisoflavonoid, flavonoid and phenolic compounds. Vietnam J. Chem..

[38] Thuy TT (2013). Phytochemical study on the plants of the antidrug medication Heantos 4. Part I. Alkaloids and other nitrogen containing compounds. Vietnam J. Chem..

[39] Thuy TT (2013). Phytochemical study on the plants of the antidrug medication Heantos 4. Part 4. Phthalides, fatty acids, oligosaccharide esters and miscellaneous compounds. Vietnam J. Chem..

[40] Anh NTH (2013). Phytochemical study on the plants of the antidrug medication Heantos 4. Part 2. Terpenoid- and steroid compounds. Vietnam J. Chem..

[41] Nesbit MA, Phillips AG (2020). Tetrahydroprotoberberines: A novel source of pharmacotherapies for substance use disorders?. Trends Pharmacol. Sci..

[42] Wang JB, Mantsch JR (2012). l-tetrahydropalamatine: A potential new medication for the treatment of cocaine addiction. Future Med. Chem..

[43] Jin GZ (1987). (–)-Tetrahydropalmatine and its analogues as new dopamine receptor antagonists. Trends Pharmacol. Sci..

[44] Ma ZZ (2008). Isoquinoline alkaloids isolated from Corydalis yanhusuo and their binding affinities at the dopamine D1 receptor. Molecules.

[45] Liu X (2012). Responses of dopaminergic, serotonergic and noradrenergic networks to acute levo-tetrahydropalmatine administration in naive rats detected at 9.4 T. Magn. Reson. Imaging.

[46] Faison SL, Schindler CW, Goldberg SR, Wang JB (2016). l-tetrahydropalmatine reduces nicotine self-administration and reinstatement in rats. BMC Pharmacol. Toxicol..

[47] Marcenac F, Jin GZ, Gonon F (1986). Effect of l-tetrahydropalmatine on dopamine release and metabolism in the rat striatum. Psychopharmacology.

[48] Seeman, P. *et al.* The cloned dopamine D2 receptor reveals different densities for dopamine receptor antagonist ligands. Implications for human brain positron emission tomography. *Eur. J. Pharmacol.* **227,** 139–146 (1992). 10.1016/0922-4106(92)90121-b 1358662

[49] Martelle JL, Nader MA (2008). A review of the discovery, pharmacological characterization, and behaivoral effects of the dopamine D2-like receptor antagonist eticlopride. CNS Neurosci. Ther..

[50] Horst NK, Jupp B, Roberts AC, Robbins TW (2019). D2 receptors and cognitive flexibility in marmosets: Tri-phasic dose–response effects of intra-striatal quinpirole on serial reversal performance. Neuropsychopharmacology.

[51] Georges F, Stinus L, Bloch B, Le Moine C (1999). Chronic morphine exposure and spontaneous withdrawal are associated with modifications of dopamine receptor and neuropeptide gene expression in the rat striatum. Eur. J. Neurosci..

[52] Fox ME, Rodeberg NT, Wightman RM (2017). Reciprocal catecholamine changes during opiate exposure and withdrawal. Neuropsychopharmacology.

[53] Tomasini C, Guidorzi R, Bianchi C, Beani L (1992). Clonidine inhibition of norepinephrine release from normal and morphine-tolerant guinea pig cortical slices. J. Neurochem..

[54] Georges F, Aston-Jones G (2003). Prolonged activation of mesolimbic dopaminergic neurons by morphine withdrawal following clonidine: Participation of imidazoline and norepinephrine receptors. Neuropsychopharmacology.

[55] Bousquet P (2001). I1 Receptors, cardiovascular function, and metabolism. Am. J. Hypertens..

[56] Altier N, Stewart J (1999). The role of dopamine in the nucleus accumbens in analgesia. Life Sci..

[57] Di Ciano P (1995). Comparison of changes in extracellular dopamine concentrations in the nucleus accumbens during intravenous self-administration of cocaine or d-amphetamine. Behav. Pharmacol..

[58] Hernandez L, Hoebel BG (1988). Food reward and cocaine increase extracellular dopamine in the nucleus accumbens as measured by microdialysis. Life Sci..

[59] Taepavarapruk P, Butts KA, Phillips AG (2014). Dopamine and glutamate interaction mediates reinstatement of drug-seeking behavior by stimulation of the ventral subiculum. Int. J. Neuropsychopharmacol..

[60] Kuczenski R, Segal DS (1997). Effects of methylphenidate on extracellular dopamine, serotonin, and norepinephrine: Comparison with amphetamine. J. Neurochem..

[61] Rowley HL (2014). Differences in the neurochemical and behavioural profiles of lisdexamfetamine methylphenidate and modafinil revealed by simultaneous dual-probe microdialysis and locomotor activity measurements in freely-moving rats. J. Psychopharm..

[62] Westerink BH, De Vries JB (1989). On the mechanism of neuroleptic induced increase in striatal dopamine release: Brain dialysis provides direct evidence for mediation by autoreceptors localized on nerve terminals. Neurosci. Lett..

[63] Hernandez L, Hoebel BG (1989). Haloperidol given chronically decreases basal dopamine in the prefrontal cortex more than the striatum or nucleus accumbens as simultaneously measured by microdialysis. Brain Res. Bull..

[64] Halbsguth C, Meissner O, Häberlein H (2003). Positive cooperation of protoberberine type 2 alkaloids from Corydalis cava on the GABA(A) binding site. Planta Med..

[65] Figueroa-Guzman Y (2011). Oral administration of levo-tetrahydropalmatine attenuates reinstatement of extinguished cocaine seeking by cocaine, stress or drug-associated cues in rats. Drug Alcohol Depend..

[66] Xi ZX (2007). Levo-tetrahydropalmatine inhibits cocaine's rewarding effects: Experiments with self-administration and brain-stimulation reward in rats. Neuropharmacology.

[67] Mantsch JR (2007). Levo-tetrahydropalmatine attenuates cocaine self-administration and cocaine-induced reinstatement in rats. Psychopharmacology.

[68] Mantsch JR (2010). Levo-tetrahydropalmatine attenuates cocaine self-administration under a progressive-ratio schedule and cocaine discrimination in rats. Pharmacol. Biochem. Behav..

[69] Kim T, Hinton DJ, Johng S, Wang JB, Choi D-S (2013). Levo-tetrahydropalmatine decreases ethanol drinking and antagonizes dopamine D(2) receptor-mediated signaling in the mouse dorsal striatum. Behav. Brain Res..

[70] Sushchyk S, Xi ZX, Wang JB (2016). Combination of levo-tetrahydropalmatine and low dose naltrexone: A promising treatment for prevention of cocaine relapse. J. Pharmacol. Exp. Ther..

[71] Yuan CS, Bieber EJ (2003). Textbook of Complementary and Alternative Medicines.

[72] Lee G, Goosens KA (2015). Sampling blood from the lateral tail vein of the rat. J. Vis. Exp..

[73] Pegg CC, He C, Stroink AR, Kattner KA, Wang CX (2010). Technique for collection of cerebrospinal fluid from the cisterna magna in rat. J. Neurosci. Methods.

[74] Sun M, Liu J, Lin C, Miao L, Lin L (2014). Alkaloid profiling of the traditional Chinese medicine *Rhizoma corydalis* using high performance liquid chromatography-tandem quadrupole time-of-flight mass spectrometry. Acta Pharm. Sin. B.

[75] Spitzer M, Wildenhain J, Rappsilber J, Tyers M (2014). BoxPlotR: A web tool for generation of box plots. Nat. Methods.

[76] Ge X, Zhang H, Zhou H, Xu Z, Bian C (1999). Experimental study of tetrahydroprotoberberines inhibiting morphine withdrawal syndromes. Chin. J. Drug Depend..

[77] Yue K (2012). The dopamine receptor antagonist levo-tetrahydropalmatine attenuates heroin self-administration and heroin-induced reinstatement in rats. Pharmacol. Biochem. Behav.

[78] Yang Z (2008). Medication of l-tetrahydropalmatine significantly ameliorates opiate craving and increases the abstinence rate in heroin users: a pilot study. Acta Pharmacol. Sin.

[79] Liu YL (2005). Effects of l-tetrahydropalmatine on locomotor sensitization to oxycodone in mice. Acta Pharmacol. Sin.

[80] Liu YL, Yan LD, Zhou PL, Wu CF, Gong ZH (2009). Levo-tetrahydropalmatine attenuates oxycodone-induced conditioned place preference in rats. Eur. J. Pharmacol.v.

[81] Ma B (2014). L-stepholidine, a natural dopamine receptor D1 agonist and D2 antagonist, inhibits heroin-induced reinstatemen. Neurosci. Lett..

[82] Wang W (2007). The effect of l-stepholidine, a novel extract of Chinese herb, on the acquisition, expression, maintenance, and re-acquisition of morphine conditioned place preference in rats. Neuropharmacology.

[83] Yue K (2014). L-Stepholidine, a naturally occurring dopamine D1 receptor agonist and D2 receptor antagonist, attenuates heroin self-administration and cue-induced reinstatement in rats. Neuroreport.

[84] Jin GZ, Zhu ZT, Fu Y (2002). (–)-Stepholidine: a potential novel antipsychotic drug with dual D1 receptor agonist and D2 receptor antagonist actions. Trends Pharmacol. Sci..

[85] Natesan S (2008). The antipsychotic potential of l-stepholidine—a naturally occurring dopamine receptor D1 agonist and D2 antagonist. Psychopharmacology.

[86] Meade JA (2015). (–)-Stepholidine is a potent pan-dopamine receptor antagonist of both G protein- and β-arrestin-mediated signaling. Psychopharmacology.

[87] Qian W (2012). Design, synthesis, and pharmacological evaluation of novel tetrahydroprotoberberine derivatives: selective inhibitors of dopamine D1 receptor. Bioorg. Med. Chem.

[88] Dong ZJ, Jin GZ, Huang HY (1995). Augmentation of dopamine release by (–)-stepholidine from rabbit and rat caudate slices. Zhongguo Yao Li Xue Bao.

[89] Sun BC, Jin GZ (1992). Effects of (–)-stepholidine on firing activity of dopamine neurons in ventral tegmental area of rats. Zhongguo Yao Li Xue Bao.

[90] Dai WL (2018). Selective blockade of spinal D2DR by levo-corydalmine attenuates morphine tolerance via suppressing PI3K/Akt-MAPK signaling in a MOR-dependent manner. Exp. Mol. Med..

