# Supplementary material for: Neural bases for attenuation of morphine withdrawal by Heantos-4: role of l-tetrahydropalmatine
Source: Sci Rep. 2020 Dec 4;10:21275. doi: 10.1038/s41598-020-78083-x (PMC7718916; doi:10.1038/s41598-020-78083-x)
Supplement: Supplementary file 1 — Supplementary Information. [file 41598_2020_78083_MOESM1_ESM.pdf]

Neural bases for attenuation of morphine withdrawal by Heantos-4: role of *l*-tetrahydropalmatine

Soyon Ahn[1], Maya O. Nesbit[1], Haiyan Zou[1], Giada Vacca[1], Peter Axerio-Cilies[1], Tran Van Sung[2] and Anthony G. Phillips[1,*]

[1]Department of Psychiatry, University of British Columbia, Vancouver, Canada V6T 2A1
[2]Institute of Chemistry, Vietnam Academy of Science and Technology, Hanoi, Vietnam
*aphillips@psych.ubc.ca

Supplementary Table S1. Basal dopamine concentration in the nucleus accumbens of morphine-treated and -naïve rats in microdialysis experiments.

Morphine treatment	Experimental groups	n	nM (mean ± s.e.m)
Treated	Experiment 1	13	1.09 ± 0.1
Naïve	Experiment 2:		
	Quinpirole+Eticlopride group	46	0.90 ± 0.06
	Quinpirole+Heantos-4 group	44	1.09 ± 0.10
Naïve	Experiment 4:		
	Quinpirole+*l*-Tetrahydropalmatine group	28	1.05 ± 0.13
	Quinpirole+*l*-Tetrahydropalmatine/Eticlopride group	27	1.44 ± 0.15
Treated	Experiment 5	20	1.02 ± 0.12

Rats were treated with morphine (10 mg/kg, *i.p.*) on Days 1-7. Dopamine concentration reflects the mean of the final three consecutive samples collected during the baseline period on Day 8.

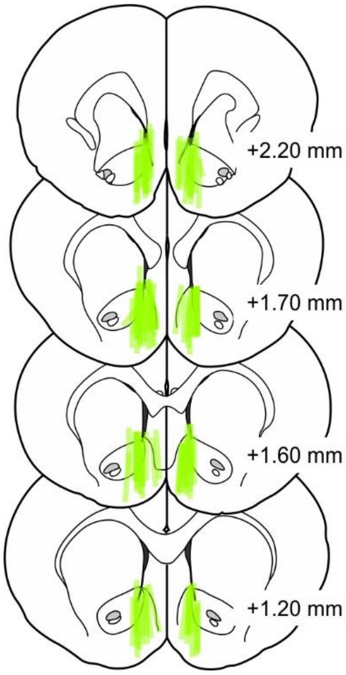

Supplementary Figure S1. Histological verification of microdialysis probe placement in the nucleus accumbens. Vertical lines represent the semi-permeable membrane portion of probes (2 mm x 340 µm OD). Distance from bregma is indicated. Drawings of coronal sections were adapted from Paxinos and Watson[1].

Supplementary Reference

1. Paxinos, G. & Watson, C. *The Rat Brain in Stereotaxic Coordinates*s (Academic Press, 1997).
